# Mindfulness-Based Crisis Interventions for patients with psychotic symptoms on acute psychiatric wards (amBITION study): protocol for a feasibility randomised controlled trial

**DOI:** 10.1186/s40814-016-0082-y

**Published:** 2016-08-05

**Authors:** Pamela Jacobsen, Emmanuelle Peters, Paul Chadwick

**Affiliations:** 1 Department of Psychology (PO 78), Institute of Psychiatry, Psychology and Neuroscience (IoPPN), King’s College London, SE5 8AF London, UK; 2 NIHR Biomedical Research Centre for Mental Health, South London and Maudsley NHS Foundation Trust, London, UK

**Keywords:** Randomised controlled trial, Crisis intervention, Inpatients, Psychosis, Psychological therapy, Mindfulness

## Abstract

**Background:**

Inpatient psychiatric care is a scarce and expensive resource in the National Health Service (NHS), with chronic bed shortages being partly driven by high re-admission rates. People often need to go to a hospital when they have a mental health crisis due to overwhelming distressing psychotic symptoms, such as hearing voices (hallucinations) or experiencing unusual beliefs (delusions). Brief talking therapies may be helpful for people during an acute inpatient admission as an adjunct to medication in reducing re-admission rates, and despite promising findings from trials in the USA, there have not yet been any clinical trials on this kind of intervention within NHS settings.

**Methods/design:**

The amBITION study is a feasibility randomised controlled trial (RCT) of a manualised brief talking therapy (Mindfulness-Based Crisis Intervention (MBCI)). Inpatients on acute psychiatric wards are eligible for the study if they report at least one positive psychotic symptom and are willing and able to engage in a talking therapy. In addition to treatment as usual (TAU), participants will be randomly allocated to receive either MBCI or a control intervention (social activity therapy (SAT)) which will be based on doing activities on the ward with the therapist. The primary objective of the study is to find out whether it is possible to carry out this kind of trial successfully within UK inpatient settings and to find out whether patients and staff find it an acceptable intervention. The secondary objective is to collect pilot data on primary and secondary outcome measures, including re-admission rates at 6-month follow-up. This will provide information on the appropriateness of re-admission as the primary outcome measure for future efficacy trials, as well as data on the acceptability and utility of the clinical self-report measures.

**Discussion:**

The results of the feasibility trial will indicate whether a subsequent efficacy pilot trial is warranted and, if so, will provide vital information for the planning of such a trial (e.g. pilot data on expected effect sizes). If future research finds that MBCI is an effective and safe intervention, then patients will benefit from access to better treatment within inpatient care which would reduce re-admission rates. This trial therefore addresses an area of urgent concern for service users, clinicians and the wider NHS.

**Trial registration:**

Current controlled trials ISRCTN37625384

## Background

People often need to go to a hospital when they have a mental health crisis due to overwhelming distressing psychotic symptoms, such as hearing voices (hallucinations) or experiencing unusual beliefs (delusions). However, inpatient care is the most costly, and over-subscribed, form of care provided by NHS mental health trusts. Mental health trusts across the United Kingdom (UK) can neither afford, nor physically accommodate, all the patients requiring admissions [1]. Reducing admission rates is therefore an area of urgent priority given on-going bed closures, with a recent study reporting a 62 % reduction in psychiatric beds nationwide from 1988 to 2008 [2]. Psychological interventions are well-established in their efficacy for reducing psychotic symptoms that have not responded adequately to pharmacological intervention [3]. However, most therapy trials in the UK have been conducted in outpatient settings, with therapy lasting approximately 6 months [4]. There is a dearth of robust evidence for the feasibility and efficacy of brief psychological interventions exclusively within acute inpatient settings. This could be due to unfounded assumptions that inpatients are always too unwell to make use of therapy or that therapy always has to be lengthy to be of any benefit. However, this may represent a missed opportunity to engage patients in psychological therapies at a critical point in the care pathway, using crisis-focused interventions. When people are admitted to a hospital at times of crisis, this can be an ideal time to offer psychological interventions as problematic thoughts, feelings and behaviours are readily accessible and the inpatient setting provides wider support. Two research studies conducted in the USA have investigated brief psychological interventions for inpatients with psychotic symptoms [5, 6]. Participants received between one and five individual sessions of an acceptance-based therapy known as acceptance and commitment therapy (ACT). Brief crisis-focused interventions target risk of future relapse and re-admission by seeking to help a person understand how their existing coping strategies have brought them into crisis and to develop skills in alternative coping strategies. ACT interventions aim to increase what is termed *psychological flexibility*, defined as “the ability to contact the present moment more fully and without needless defence” [7]. Patients’ existing coping strategies are often lacking in psychological flexibility, relying instead heavily on experiential avoidance (i.e. attempts to avoid unwanted thoughts, feelings or sensations) [8]. For example, a study of 50 patients experiencing voices in the context of a psychotic illness found that being less accepting towards internal experiences was positively associated with behavioural attempts to resist voices [9]. For example, people may cope with unpleasant auditory hallucinations by drinking alcohol or using illicit drugs in an attempt to block them out. Someone experiencing persecutory delusions may choose to avoid the anxiety they feel when they go out in public by isolating themselves at home. These behaviours not only stop the person from being able to function normally in their everyday life but also increase the risk of serious self-neglect and re-admission. ACT therefore aims to help people to accept symptoms, rather than trying to avoid or eliminate them, and to defuse or step back from them, while promoting behaviours which are consistent with the person’s underlying values and goals in life [7]. This acceptance-based approach is also consistent with Chadwick’s model of mindfulness for psychosis, in which people are taught skills in relating mindfully to psychotic symptoms, as an alternative to either experiential avoidance or simply getting lost in struggle and rumination [10].

The two USA studies found that the intervention was successful in reducing re-admission rates at 4-month follow-up. Bach and Hayes reported that the re-hospitalisation rate of the ACT group was half that of the treatment as usual (TAU) group (20 vs. 40 % respectively), a statistically significant difference. Gaudiano and Herbert (2006) reported the same trend (28 % ACT vs. 45 % TAU respectively), but these results did not reach statistical significance. It is not yet known whether such brief crisis-focused interventions would translate effectively to NHS inpatient care in the UK. As brief crisis-focused psychological interventions have not been subject to controlled trials in the UK, this study will be a feasibility trial providing valuable data to inform possible later efficacy pilot trials (BrIef Talking therapIes ON wards (amBITION) study). A brief, manualised therapy (Mindfulness-Based Crisis Intervention (MBCI)) will be compared with an active control condition (social activity therapy (SAT)) to help account for non-specific elements of therapy, such as having individual attention from an empathic therapist.

The primary objective of the study is to find out whether it is possible to carry out this kind of trial successfully within inpatient settings and to find out whether patients and staff find it an acceptable intervention. The secondary objective is to collect pilot data on primary and secondary outcome measures.

## Methods/design

### Study design and timeline

**Fig. 1:**
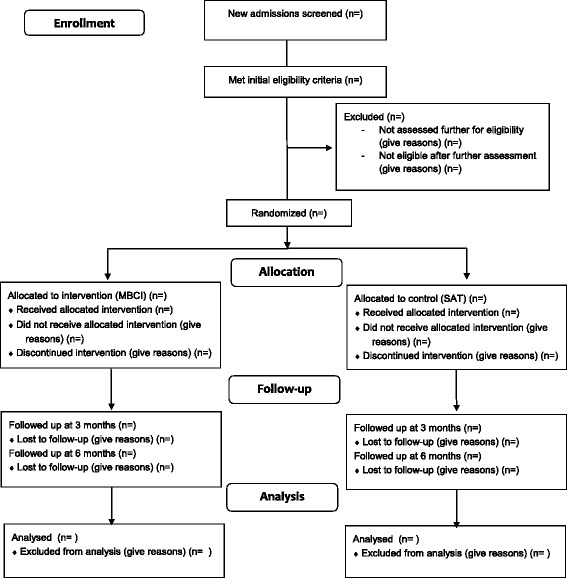
Study plan

This study is a single-centre, parallel-groups, feasibility randomised controlled trial. Trial procedures and the assessment schedule are shown in the study plan (Fig. 1). In brief, service use data (re-hospitalisation rate, use of crisis team, relapse rate) will be collected using case note review at 3- and 6-month follow-up. Self-report clinical measures will be taken at baseline, post-therapy and at 3-month (mid-point) and 6-month follow-up after discharge (end-point). The 3-month mid-point follow-up was included in order to minimise missing data arising from loss to follow-up and to provide more detailed information on symptom change in the short-term after discharge. The service use data will provide information on the appropriateness and sufficiency of re-admission as the primary outcome measure for future efficacy trials or whether an additional primary outcome would be indicated (e.g. relapse rate from case note review). The trial will also provide important data on the acceptability and utility of the self-report clinical measures, for example, whether participants are willing and able to complete the measures and whether they show sensitivity to change over time.

### Study population

#### Inclusion criteria

i) Aged 18 or above
ii) Current psychiatric inpatient on a working-age adult ward
iii) Diagnosis of schizophrenia-spectrum disorder or psychotic symptoms in the context of an affective disorder (ICD-10 codes F20-39; [11])
iv) Reports at least one current positive psychotic symptom (scores >1 on frequency on self-report symptom scale)
v) Able to give informed consent to participate in trial, as assessed by consultant psychiatrist/responsible clinician
vi) Willing and able to engage in psychological therapy

#### Exclusion criteria

i) Established diagnosis of learning disability or major cognitive impairment arising from any underlying medical condition (e.g. head injury, neurological disorder) resulting in significant functional impairment
ii) Unable to engage in a talking therapy in English or to complete simple written questionnaires in English
iii) Primary diagnosis of substance misuse
iv) Lacks capacity to consent to participation in research trial
v) Unable to take part in individual therapy due to risk of aggression/violence
vi) Mental state precludes possibility of engaging in a talking therapy, e.g. significant thought disorder

#### Recruitment, randomisation and blinding

Patients will be recruited from acute inpatient psychiatric wards from a large mental health trust in South London, serving a local population of 1.1 million people, with approximately 6000 acute inpatient admissions a year. Potentially eligible patients will be identified by their inpatient care team and will be approached to take part by the researcher with permission of their inpatient Consultant Psychiatrist and primary nurse. Patients may take part in the trial if they are admitted under a section of the Mental Health Act (MHA) so long as they are deemed to have retained capacity to consent to participation in research. Further eligibility screening by reference to electronic clinical notes will be conducted with written consent from patients who have been approached and are potentially interested in participating. Patients will be given a copy of the brief patient leaflet at this point to introduce them to the main aims of the study. Once the researcher has confirmed the patient’s eligibility, she will approach the patient again to give them a copy of the full patient information sheet and to talk it over with them and explain the study further. Patients will be given sufficient time (at least until the next day) to read over the information, think it over, ask questions and to discuss their participation with anyone they may wish to (e.g. primary nurse, family member). After giving informed consent, eligible participants will first complete baseline measures and then be randomised using a computerised service at the Kings Clinical Trials Unit (KCTU). Due to the nature of the intervention, blinding of participants and therapist is not possible. The participant’s inpatient and community care team will however be blinded to treatment allocation, as far as possible. Conservative measures will be used such as not referring to any content of the therapy sessions in clinical notes and conducting all therapy sessions in a private room on the ward. The two therapies will be referred to by neutral labels in all participant and staff literature (therapy 1 vs. therapy 2) in order to promote equal treatment credibility between the conditions. Block randomisation will be used, with randomly varying block sizes to ensure allocation concealment. As this is a feasibility trial, the primary outcomes relate to feasibility data rather than clinical outcomes. PJ will be primarily responsible for gathering all trial data and will not be blinded to treatment condition, but some follow-up data may also be collected wherever possible by appropriately trained staff independent of the clinical team (e.g. research nurse, postgraduate students). The study will be conducted in line with Good Clinical Practice (GCP) guidelines for clinical trials [12]. The data management plan includes standard procedures such as the use of anonymous identification codes.

#### Sample size

A power calculation to determine a sample size is not appropriate for a feasibility trial, as the purpose of the trial is not to establish efficacy. However, the data from this trial could be used to inform a sample size calculation for a later efficacy pilot trial. The target recruitment for this feasibility trial will be *N* = 60 (30 in each arm).This was determined with reference to existing studies in the field and is consistent with good practice recommendations for feasibility/pilot studies [13, 14].

### Description of therapies

Therapy sessions in both conditions will be delivered on an individual basis in a private room on an inpatient ward. The trial therapist in both conditions will be PJ, who is a Clinical Psychologist registered with the UK Health and Care Professions Council (HCPC) and has expertise in cognitive behavioural therapy for psychosis (CBTp) and mindfulness interventions as well as experience of working in acute settings. Although not matched on a case by case basis, therapy sessions in both conditions will range from one to five sessions, depending on length of admission, with the frequency of sessions adjusted as needed between a minimum of weekly and maximum of daily. All sessions will follow a stand-alone, self-contained format in order to accommodate unpredictable lengths of stay and unexpected discharges. Participants in the trial will continue to receive treatment as usual (TAU) both during their inpatient admission and post-discharge. In practice, this may include medication, attendance at activity and/or therapy groups, individual therapy sessions and family therapy sessions.

#### Mindfulness-Based Crisis Interventions (MBCI)—experimental intervention

MBCI was developed in line with the model of mindfulness for psychosis proposed by Chadwick [10]. People who experience positive psychotic symptoms (e.g. voices, paranoid thoughts) often respond by trying to avoid experiences (experiential avoidance) or at the other end of the spectrum, by getting lost in engaging with them (rumination, confrontation). Mindfulness offers an alternative way of responding, with acceptance and non-judgemental awareness in each moment, allowing psychotic symptoms to move in and out of awareness without the person getting caught up in struggling against them. The treatment protocol for the current trial was adapted for use within an acute crisis setting, partly based on PJ’s clinical experience of working within inpatient settings and in consultation with ACT experts in the USA, including the lead author of one of the key inpatient trials [6].

There are three key components to be included in each session:

i. Developing mindfulness skills (guided practice)
ii. Making sense of crisis using mindfulness model
iii. Identifying values and setting goals

A typical session will start with a 5-min mindfulness practice, including frequent guidance that includes reference to psychotic experience and uses everyday, concrete language. The therapist will then move on to developing a collaborative understanding with the participant of what has brought them to a hospital on this occasion, focussing on how they usually try to cope with difficult voices, thoughts, feelings and experiences and how well these strategies are working for them. Given the time constraints, therapists will share a formulation and example strategies (following Chadwick [10]), asking the participant to connect with and provide examples of his or her own habitual reactions. In line with the formulation, the therapist will highlight the participant’s attempts to either block out, suppress or otherwise escape from unwanted internal experiences or reactions that mean getting caught up in struggling with internal experiences (rumination, fighting). Mindfulness is located as a middle way between these two reactive styles. Finally, the therapist will work with the participant to identify their values (e.g. family, work, health, society) and discuss specific behavioural goals consistent with these values. Participants are then helped to set a small, achievable goal for homework at the end of each session which can be reviewed at the beginning of next session, where possible. In preparation for discharge, longer-term goals can also be identified (e.g. starting a college course) and will be shared with the community care team at the end of therapy, to act as a bridge to carrying on the recovery process in the community.

#### Social activity therapy (SAT)—control intervention

This control condition is taken from the PICASSO trial of CBTp for people with psychosis and a history of violence and was conducted partly on inpatient wards [15]. SAT involves collaboratively working with the participant to identify activities they enjoy and which they can engage in during sessions and between sessions as they wish (e.g. board games, puzzles). The aim is to provide a supportive environment with a therapist using non-specific aspects of therapy (e.g. agenda setting, collaboration, feedback, empathy). The therapist aims to keep the sessions activity focussed and to be supportive, collaborative and empathic without employing any therapy techniques specific to any model of therapy, including CBTp or mindfulness-based therapies.

#### Treatment fidelity

The trial therapist will receive regular supervision from an independent clinical supervisor with expertise in acute care and mindfulness-based approaches. Therapy sessions will be audio-taped with participant consent (the proportion of participants who consent to audio-recording will also be recorded and reported as part of the trial outcomes). A sample of therapy sessions will be assessed by a blinded and independent rater for therapy fidelity. An adherence and competency scale for the trial has been developed for this purpose, based on existing scales from other therapy trials [15–17] and relevant theoretical papers and therapy manuals [7, 10, 18].

### Outcome measures

#### Primary objective—feasibility/acceptability data

1. Number of eligible participants identified over study period
2. Total numbers recruited into trial and recruitment rate (benchmark of 80 % of target)
3. Proportion of participants who drop out during the intervention stage
4. Range and average number of sessions completed (including number of sessions attended as a proportion of those offered)
5. Reasons for participants dropping out during the intervention stage
6. Number lost to follow-up and reasons (benchmark of less than 20 % to be set in line with previous studies)
7. Any unexpected adverse effects of participating in the trial

#### Qualitative data on acceptability

1. Participant feedback on trial procedures, randomisation and credibility of two therapies
2. Staff feedback on trial procedures, recruitment strategies and blinding procedures

At the end of the study, participants will be asked if they are willing to give feedback on trial procedures and therapy by way of a follow-up interview or focus group. Participation will be optional. The feedback interviews/focus groups will be conducted by an appropriately trained service user researcher. Staff from the inpatient units where patients were recruited will also be invited to give feedback on the trial via interview or focus group and will be asked to give informed written consent. Interviews and focus groups will be audio-recorded, with written consent from all participants. Qualitative data will be analysed using thematic analysis [19], which is a commonly used approach within applied health research. The data will be initially coded line-by-line to identify emergent themes, which will then be grouped together into larger themes and sub-themes. The data will be coded by at least two people, in order to allow some degree of inter-rater reliability.

#### Secondary objective—pilot data

Pilot outcome measures (service use and clinical measures) will be collected, as detailed in Table 1. A cost-effectiveness analysis of the intervention is outside the scope of this feasibility study; however, the service-use data collected would be relevant to the future assessment of economic costs. This is in addition to data on therapy costs which will be collected, such as the average number of sessions received per participant.

**Table 1:** Summary of outcome measures

Pilot data—inpatient/crisis service use			
Outcome		Method	Time period
Primary outcome:			
1) Re-hospitalisation (≥1 OBD)		Clinical notes	Discharge—3- and 6-month follow-up
Secondary outcomes:			
2) Time to re-admission (days)		Clinical notes	Discharge—3- and 6-month follow-up
3) Total number of OBDs		Clinical notes	Discharge—3- and 6-month follow-up
4) Episodes of care with crisis/home treatment team		Clinical notes	Discharge—3- and 6-month follow-up
5) Contact with CMHT (number of meetings/contact with CMHT including care co-ordinator)		Clinical notes	Discharge—3- and 6-month follow-up
6) Reference to therapy goal which was shared with team		Clinical notes	Discharge—3- and 6-month follow-up
7) Relapse rate		Clinical notes	Discharge—3- and 6-month follow-up
Pilot data—clinical measures			
Construct assessed	Questionnaire	Method	Time points
Credibility of therapy	1) Therapy credibility	Self-report	Baseline only (immediately post-randomisation)
In the moment rating of stress and interference from symptoms and hope for the future	2) Stress bubbles	Self-report	At the beginning and end of every therapy session
Frequency, distress and believability of most distressing symptom	3) Self-ratings of psychotic symptoms (based on Bach and Hayes, 2002; Gaudiano and Herbert, 2006)	Self-report	Baseline, end of therapy, 3-month mid-point and 6-month follow-up
Mood—depression, anxiety and stress	4) DASS-21 (Depression, Anxiety and Stress Scale; Lovibond and Lovibond, 1995)	Self-report	Baseline, end of therapy, 3-month mid-point and 6-month follow-up
Self-defined recovery	5) QPR (Questionnaire about the Process of Recovery; Neil et al 2009)	Self-report	Baseline, end of therapy, 3-month mid-point and 6-month follow-up
Voices (incl. frequency, distress, interference and compliance)	6) HPSVQ (Hamilton Program for Schizophrenia Voices Questionnaire; Van Lieshout and Goldberg, 2007)	Self-report	Baseline, end of therapy, 3-month mid-point and 6-month follow-up
Mindfulness	7) SMQ (Southampton Mindfulness Questionnaire; Chadwick et al, 2008)	Self-report	Baseline, end of therapy, 3-month mid-point and 6-month follow-up

*OBD* occupied bed day, *CMHT* community mental health team

#### Description of clinical measures

1. Therapy credibility
  Immediately after randomisation, participants will be read a brief description of the therapy they have been assigned to. They will then be asked to rate on a scale from 0 (not helpful at all) to 10 (extremely helpful) how helpful they think this therapy sounds.
2. Stress bubbles
  The use of within-session measures can be helpful in measuring change in brief interventions, by capturing small shifts in key processes that may occur over the course of a therapy session. Stress bubbles are a form of visual analogue scale, with six bubbles gradually increasing in size from “not at all” (1) to “extremely” (6). Respondents rate three items (stress, interference from symptoms and hope for the future) at the beginning and end of every session. These unpublished scales have been successfully used in a previous study of mindfulness interventions for psychosis [20].
3. Self-ratings of psychotic symptoms
  This is a self-report scale which asks respondents to rate their psychotic symptoms (voices and/or distressing beliefs) on a scale of 1–7 (frequency) and 0–10 (distress and believability). These scales were used in the ACT inpatient trials [5, 6] and were found to be easy for participants to complete and showed sensitivity to change over time.
4. Depression, Anxiety and Stress Scales (DASS-21) [21]
  The DASS-21 is a short-form version of the original 42-item DASS comprising seven items on each of the three sub-scales for depression, anxiety and stress. It is a self-report scale with respondents scoring each item on a four-point scale from 0 (never) to 3 (almost always). The DASS-21 has been well-validated in both clinical [22] and non-clinical samples [23]. The DASS-21 is particularly suitable for this study, being relatively quick and easy to complete, and has been shown to have good internal consistency and convergent validity in an acute psychiatric population [24] and is suitable for use with people experiencing psychotic symptoms [25].
5. Questionnaire about the Process of Recovery (QPR) [26]
  The QPR is a 22-item self-report measure based on service user accounts of the process of recovery from psychosis. It has two sub-scales assessing both intrapersonal and interpersonal processes in recovery. Each item is rated on a five-point scale from 0 (disagree strongly) to 4 (agree strongly). Neil et al. [26] report that the scale has good internal consistency, construct validity and reliability.
6. Hamilton Program for Schizophrenia Voices Questionnaire (HPSVQ) [27]
  The HPSVQ is a 13-item self-report measure in which respondent rate the first nine items on a five-point Likert scale from zero (lowest severity) to four (highest severity). The total score of these nine items is intended to indicate the severity of auditory verbal hallucinations and includes items on frequency, distress and interference with daily activities. There are an additional four qualitative items, not included for the purposes of this study. Kim et al. [28] reported high test-retest reliability and good convergent validity with established clinician-rated scales (PSYRATS-AH [29]; PANSS [30]) when used in a clinical sample of people with a diagnosis of schizophrenia.
7. Southampton Mindfulness Questionnaire (SMQ) [31]
  The SMQ is a 16-item self-report measure designed to assess mindfulness of difficult thoughts and images. Each item is scored on a seven-point scale ranging from 0 (totally agree) to 6 (disagree totally). The SMQ has been validated in a clinical sample of people experiencing distressing psychotic symptoms. Chadwick et al. [31] report that the SMQ has good internal reliability and shows convergent reliability with other established mindfulness scales (e.g. MAAS; [32]).

### Service user involvement

Service user involvement has been the key to the development of the trial protocol through consultation with local groups. Service users who have been consulted have supported the aims of the trial enthusiastically because they report feeling the provision of talking therapies on inpatient units is a very neglected and under-researched part of mental health care. A Service User Advisory Group (SUAG) has also been convened for the purposes of providing further consultation over the course of the study. Members of the SUAG will provide input to the Trial Steering Committee (TSC), in addition to taking the lead on carrying out feedback interviews and running focus groups with participants after the trial has ended.

### Analysis plan

Descriptive statistics will be reported for the key outcomes on the feasibility data (including mean averages, standard deviations and ranges where appropriate). Flow through the trial will also be presented in a standard CONSORT diagram, showing number approached to participate, number randomised, drop-outs before the end of treatment and numbers retained in the trial at 3- and 6-month follow-up. Pilot data on the primary outcome of re-hospitalisation at 6-month follow-up will be analysed using survival analysis. The proportion *n* (%) of patients readmitted at 3 and 6 months will be reported, with the difference in time to re-admission between intervention and control groups being formally compared using Kaplan-Meier/Log rank survival analysis. Odds ratios with 95 % confidence intervals will be calculated and used to provide an indicator of measurement precision. This will help provide information on the appropriateness of re-admission as the primary outcome measure for future trials. In order to provide data for future sample size calculations, pilot data on clinical measures will be analysed using the general linear model, co-varying for baseline score and treatment condition. All analyses will be done on an intention-to-treat principle, in consultation with the KCTU. As this is a feasibility study, it is not powered to detect treatment efficacy and accordingly all hypothesis testing should be treated as preliminary and interpreted with caution.

## Discussion

This protocol describes the first RCT of a brief talking therapy for psychosis (MBCI) designed specifically for delivery in acute inpatient settings in the UK. It builds on encouraging pilot trials from the USA which indicate that brief ACT interventions during inpatient admissions may help people to stay out of hospital for longer after discharge [5, 6]. Service users consistently report they do not have good enough access to talking therapies during inpatient admissions, although this is something they rate as a high priority [33, 34]. This research proposal therefore addresses an area of urgent concern for service users, as well an area of clinical and economic concern for the NHS, given the high cost of inpatient care. The so-called bed crisis in UK psychiatric acute care has been well-publicised recently and unfortunately shows no signs of abating. This crisis has led the Royal College of Psychiatrists to establish an independent Commission to review the provision of acute inpatient psychiatric care for adults in the UK, in response to widespread concern about whether there are sufficient beds available [35]. Providing high-quality care during an inpatient admission may help to reduce the demand for inpatient beds by reducing re-admissions rates. As well as the economic costs of “failed” discharges leading to rapid re-admission, there is of course a great personal and social cost to such failures in mental health care. Service users often report a psychiatric admission as a highly distressing, disruptive and stigmatising experience and one to be avoided at all cost [36].

### Conclusion

In summary, the results of this feasibility trial will indicate whether a subsequent efficacy pilot RCT is warranted and, if so, will provide vital information for the planning of such a trial. If future research finds that MBCI is an effective and safe intervention, then patients will benefit from access to better treatment within inpatient care which could help them stay out of hospital for longer after discharge. This would also help NHS mental health trusts to deliver more cost-effective inpatient care as savings would be made over the longer-term due to reduced service use by patients.

### Trial status

In preparation.

## Abbreviations

ACT, acceptance and commitment therapy; CBTp, cognitive behavioural therapy for psychosis; CMHT, community mental health team; DASS-21, Depression, Anxiety and Stress Scales-21; HCPC, Health and Care Professions Council; HPSVQ, Hamilton Program for Schizophrenia Voices Questionnaire; ICD-10, International Statistical Classification of Diseases and Related Health Problems, tenth edition; KCTU, King’s Clinical Trials Unit; MBCI, Mindfulness-Based Crisis Interventions; MHA, Mental Health Act; NHS, National Health Service; PANSS, Positive and Negative Syndrome Scale; PSYRATS-AH, Psychotic Symptom Rating Scales—Auditory Hallucinations; RCPsych, Royal College of Psychiatrists; RCT, randomised controlled trial; REC, Research Ethics Committee; SAT, social activity therapy; SMQ, Southampton Mindfulness Questionnaire; SUAG, Service User Advisory Group; TAU, treatment as usual; TSC, Trial Steering Committee; UK, United Kingdom; QPR, Questionnaire about the Process of Recovery
